# Kikuchi–Fujimoto Disease: A Rare Etiology Behind Pediatric Cervical and Supraclavicular Lymphadenopathy: A Case Report

**DOI:** 10.3390/reports8030182

**Published:** 2025-09-18

**Authors:** Maria Rogalidou, Vasileios Xydis, Kalliopi Stefanaki, Nikolaos Chaliasos, Ekaterini Siomou, Alexandros Makis

**Affiliations:** 1 Paediatrics Department, University Hospital of Ioannina, 45500Ioannina, Greece; nchaliasos@gmail.com (N.C.); eksiomou@uoi.gr (E.S.); amakis@cc.uoi.gr (A.M.); 2 Division of Gastroenterology & Hepatology, First Department of Paediatrics, National and Kapodistrian University of Athens, ‘Agia Sophia’ Children’s Hospital, 11527 Athens, Greece; 3 Radiology Department, University Hospital of Ioannina, 45500 Ioannina, Greece; vgxydis@gmail.com; 4 Pathology Department, “Agia Sophia” Children’s Hospital, 11527 Athens, Greece; kalstef@otenet.gr

**Keywords:** cervical lymphadenopathy, supraclavicular lymphadenopathy, children, Kikuchi-Fujimoto disease

## Abstract

**Background and Clinical Significance:** Cervical lymphadenopathy is a common condition in children, most often caused by reactive hyperplasia due to viral infections, followed by bacterial infections and, less commonly, malignancies. Supraclavicular lymphadenopathy in children warrants thorough evaluation due to its higher association with malignancy compared to anterior cervical lymphadenopathy. Kikuchi–Fujimoto disease (KFD) is a rare, benign, and self-limiting condition characterized by cervical lymphadenopathy, predominantly affecting young adults—especially Asian women—and is rarely observed in children. We present a case of a 14-year-old girl with cervical and supraclavicular lymphadenopathy diagnosed with KFD. **Case Presentation:** A previously healthy 14-year-old girl was admitted with a 20-day history of cervical and supraclavicular lymphadenopathy, fever, and 5 kg weight loss. Laboratory investigations revealed leukopenia and lymphopenia, with a weakly positive ANA titer (1:160) and no other significant abnormalities. Extensive infectious workup was negative. Cervical ultrasound showed multiple enlarged, hypoechoic, rounded lymph nodes. CT imaging revealed paraaortic lymphadenopathy without additional findings. Due to the persistence of lymphadenopathy and inconclusive workup, a lymph node biopsy was performed to rule out malignancy. Histopathology was consistent with Kikuchi–Fujimoto disease. **Conclusions:** This case highlights a rare pediatric presentation of KFD, particularly notable for supraclavicular lymphadenopathy. It underscores the importance of considering a broad differential diagnosis in persistent lymphadenopathy, including rare conditions such as Kikuchi–Fujimoto disease.

## 1. Introduction and Clinical Significance

Cervical lymphadenopathy is a common condition in children, affecting up to 90% of those aged 4 to 8 years [1]. The most frequent cause is reactive hyperplasia secondary to viral infections, followed by bacterial infections and, more rarely, malignancies [2]. Acute bilateral cases are typically associated with viral upper respiratory tract infections or streptococcal pharyngitis, whereas acute unilateral cases are more often caused by streptococcal or staphylococcal infections [3]. Subacute or chronic lymphadenopathy may result from cat scratch disease, mycobacterial infections, or toxoplasmosis [3].

Supraclavicular lymphadenopathy in children is particularly concerning, as it is more frequently associated with malignancy than anterior cervical lymphadenopathy [4].

The diagnostic approach includes a thorough clinical evaluation, relevant laboratory investigations, and imaging studies, with ultrasonography increasingly employed as the initial modality [5]. Fine-needle aspiration cytology (FNAC) may assist in diagnosis; however, in cases of persistent lymphadenopathy, excisional biopsy is often warranted, as histopathological examination remains the gold standard [6].

Kikuchi–Fujimoto disease (KFD), also known as histiocytic necrotizing lymphadenitis, is a rare, benign, and self-limiting disorder characterized by subacute necrotizing lymphadenopathy, most commonly in the cervical region. While KFD primarily affects young adult women, it can also occur in children, in whom clinical features may differ [7]. In pediatric populations, males may be more frequently affected than females, contrary to the adult demographic. Children with KFD often present with more prominent systemic symptoms, including fever, rash, and tender lymphadenopathy. Leukopenia is commonly reported in both children and adults [7].

KFD can mimic more serious conditions such as tuberculosis or lymphoma, making early and accurate diagnosis essential. Although its etiology remains unclear, both infectious and autoimmune mechanisms have been proposed [8]. Diagnosis is confirmed histologically, with characteristic features including paracortical areas of apoptotic necrosis, karyorrhectic debris, and a proliferation of histiocytes, plasmacytoid dendritic cells, and CD8+ T lymphocytes [8]. Histopathologic differentiation from systemic lupus erythematosus (SLE) and non-Hodgkin lymphoma is critical [9].

We report a rare case of Kikuchi–Fujimoto disease in a 14-year-old girl who presented with prolonged cervical and supraclavicular lymphadenopathy—an exceptionally unusual manifestation in pediatric patients. To our knowledge, this is the first documented pediatric case presenting with supraclavicular lymphadenopathy, a feature that is itself rare even among adult cases of KFD.

## 2. Case Presentation

A previously healthy 14-year-old girl was admitted to the Pediatric Department of the University Hospital of Ioannina, Ioannina, Greece, with a one-month history of cervical and supraclavicular lymphadenopathy, predominantly on the left side. Approximately 20 days prior to admission, she developed fever (maximum 39 °C), occurring every six hours for the first ten days and every twelve hours over the subsequent ten days. Additional symptoms included fatigue, transient pain in both hip joints, and unintentional weight loss of approximately 5 kg.

Her past medical history was unremarkable. Family history revealed a probable diagnosis of systemic lupus erythematosus (SLE) in her mother at age 30, made in the United States; however, her mother had been asymptomatic and off treatment for the past ten years.

**Figure 1:**
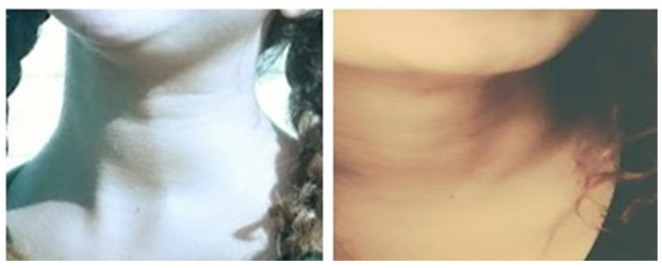
Cervical and supraclavicular lymphadenopathy.

On physical examination, the patient was febrile (38.5 °C), notably pale, but in generally good condition. Inspection revealed visible cervical and supraclavicular lymphadenopathy (Figure 1).

On palpation, the lateral cervical lymph nodes were firm, mobile, moderately tender, and measured up to ~3 cm in diameter. Smaller cervical nodes were mobile and non-tender. Supraclavicular lymph nodes—more prominent on the right—were hard, fixed, and painless. Small axillary lymph nodes were barely palpable. No other lymph node groups were enlarged. Abdominal examination revealed no organomegaly, and findings from other systemic examinations were unremarkable.

Laboratory and Imaging Investigations

Initial laboratory investigations showed a mildly elevated erythrocyte sedimentation rate (ESR) of 29 mm/h (normal range: 0–20 mm/h), and borderline hemoglobin for age and sex (12.8 g/dL; NR: 12.4–16.4 g/dL). Leukocyte counts, inflammatory markers, and other routine parameters were within normal limits.

Abdominal ultrasonography was consistent with prior imaging studies. Abdominal MRI revealed multiple splenic nodules with signal intensity similar to the splenic parenchyma, of unclear etiology (Figure 1).

Due to the broad differential diagnosis for lymphadenopathy and fever of unknown origin, an extensive workup was undertaken. The laboratory results appear in Table 1.

Ophthalmologic evaluation: normal anterior segment.

Imaging

**Figure 2:**
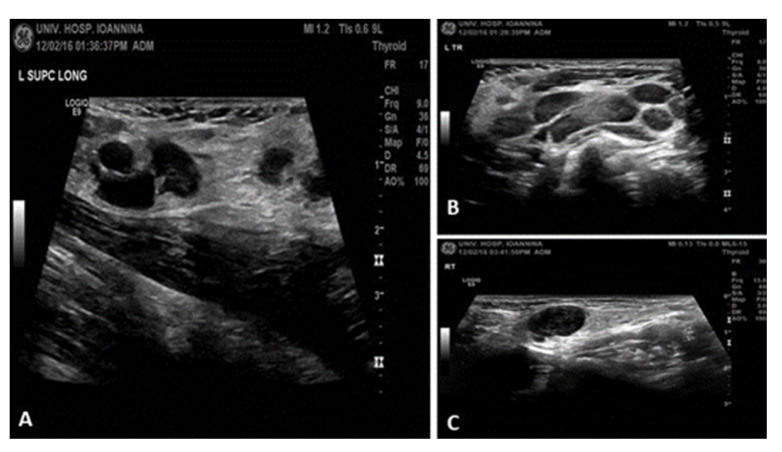
Ultrasound findings: (**A**) In the supraclavicular regions, multiple enlarged lymph nodes are also revealed that locally converge. Some of them appear hypoechoic and rounded; the largest is on the left with a maximum diameter of ~1.3 cm. (**B**,**C**) In the cervical regions and within the parotid glands, more commonly on the left, multiple enlarged lymph nodes of maximum diameter up to 2 cm are observed, which in some areas converge with an oval morphology and normal architecture.

Ultrasound: multiple enlarged, hypoechoic, rounded cervical and supraclavicular lymph nodes (Figure 2).

**Figure 3:**
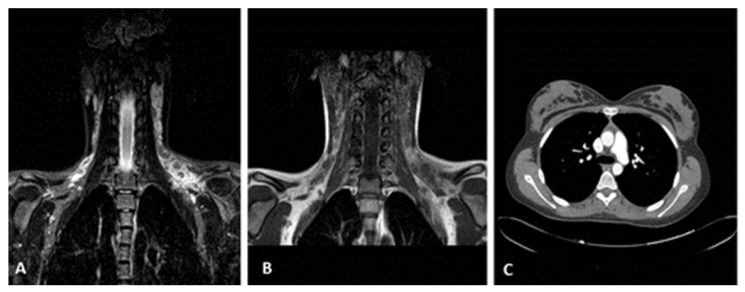
Radiological findings: (**A**,**B**) MRI in the cervical and supraclavicular areas, mainly on the left; multiple lymph nodes are observed that converge in some areas. Some of them, being rounded off, show an intermediate signal on the T1 sequence and a low signal on the STIR, while on the diffusion sequences, they show a restriction. The perilymph node tissue shows an intense high signal on fat suppression sequences to the left, while on T1 sequences, it shows inhomogeneous high signal and increased diffusion. (**C**) CT scan of the thorax and abdomen: no obvious pathological findings; a number of lymph nodes up to 7 mm in diameter are observed paraaortically and in the mesentery. Central tracheobronchial tree free, no pathologically sized lymph nodes in the hilar and mediastinum.

MRI (cervical/supraclavicular region): multiple hypoechoic, rounded lymph nodes (Figure 3A,B).

Treatment and Outcome

The patient was started on empiric broad-spectrum antibiotics. Fever was resolved by the fourth day of hospitalization, and she remained afebrile thereafter. Due to the persistence of lymphadenopathy, an excisional biopsy was performed.

**Figure 4:**
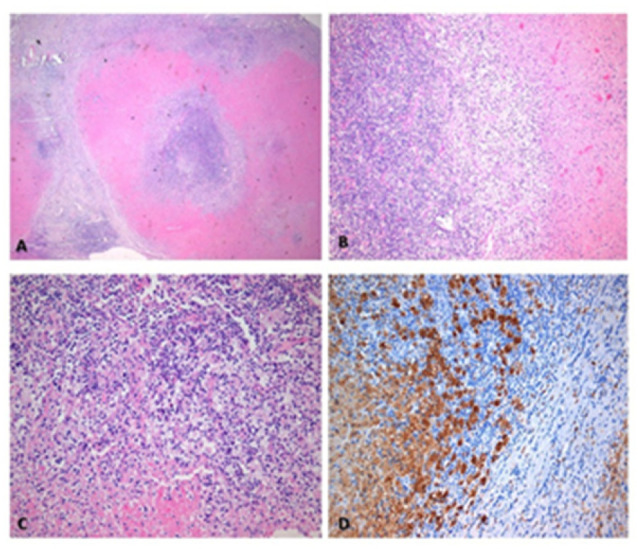
Histopathological findings: (**A**) hematoxylin–eosin staining showing extensive apoptotic necrosis without abscess formation, with surrounding histiocytes and preservation of lymph node tissue; (**B**) apoptotic necrosis and mast cells at higher magnification; (**C)** foamy histiocytes of proximal necrosis without epithelioid granuloma; (**D**) CD68 highlighting this mast cell marker in residual areas around necrosis.

Histopathological examination of lymph node tissue (0.6–1.5 cm) revealed necrotizing, histiocytic, non-suppurative lymphadenitis—predominantly in the necrotic phase—consistent with Kikuchi–Fujimoto disease (Figure 4).

Following the diagnosis, the patient was started on ibuprofen, with excellent clinical response. Cervical and supraclavicular lymphadenopathy gradually resolved, and her appetite improved.

**Figure 5:**
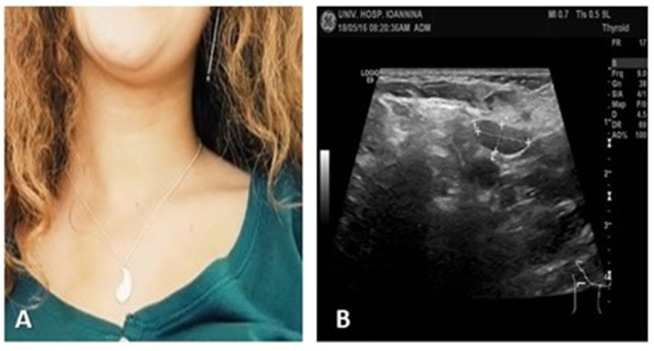
(**A**) Patient in follow-up, without lymphadenopathy; (**B**) ultrasound: 2 small lymph nodes with normal architecture and echo structure.

At one-month follow-up, the patient was asymptomatic (Figure 5A), with improved appetite and weight gain. There were no palpable cervical or supraclavicular lymph nodes, and ultrasound findings were near normal (Figure 5B). Leukopenia had resolved, although mild lymphopenia persisted for several months.

She was advised to undergo regular clinical follow-up—initially monthly, then every three months—and annual immunologic evaluations, or earlier in the event of symptom recurrence.

### Figures and Tables 

They are included in the main text.

## 3. Discussion

This case highlights an unusual presentation of Kikuchi–Fujimoto disease (KFD) in a pediatric patient, with supraclavicular lymphadenopathy as a prominent feature—a finding rarely reported, particularly in children. In pediatric clinical practice, supraclavicular lymphadenopathy warrants heightened concern due to its strong association with malignancy, compared to anterior cervical lymphadenopathy [4]. While most cases of cervical lymphadenopathy in children are reactive or infectious in origin, persistent, non-resolving supraclavicular lymph nodes, especially when firm and immobile, should raise suspicion for more serious conditions such as lymphoma or mediastinal pathology.

KFD, also known as histiocytic necrotizing lymphadenitis, is a rare, self-limiting disorder of uncertain etiology. It predominantly affects young women in their second or third decade of life and is far more commonly described in adults than in children. Pediatric presentations of KFD, especially in Western populations, are rare and often present diagnostic challenges due to nonspecific and sometimes alarming clinical features [10].

Although KFD typically presents in young adult females, pediatric cases have been reported, albeit infrequently. These often manifest with fever and lymphadenopathy, usually involving the posterior cervical lymph nodes. Supraclavicular involvement in pediatric cases is exceptionally rare. In a large series of pediatric KFD patients, supraclavicular lymphadenopathy was not identified as a typical feature [11].

Our patient presented with persistent cervical and supraclavicular lymphadenopathy, prolonged fever, weight loss, leukopenia, and lymphopenia. These nonspecific symptoms significantly overlapped with other potential diagnoses, including infectious mononucleosis, tuberculosis, autoimmune diseases such as systemic lupus erythematosus (SLE), and hematologic malignancies [12,13].

The positive antinuclear antibody (ANA) at a titer of 1:160 raised concerns about a possible autoimmune process, particularly SLE, which can present with similar features and has been known to co-exist with or evolve from KFD [13]. However, the absence of other serologic markers or clinical manifestations of SLE, along with the lack of systemic organ involvement, argued against this diagnosis in our case.

Imaging studies—including ultrasound and MRI—revealed multiple hypoechoic, rounded lymph nodes, and CT scans showed paraaortic and mesenteric lymphadenopathy, raising initial concerns for lymphoma. Due to the persistence of lymphadenopathy and systemic symptoms, an excisional biopsy was appropriately performed to establish a definitive diagnosis.

Histopathological examination remains the gold standard for diagnosing KFD. In our case, biopsy revealed classic features of the disease, including patchy paracortical necrosis, karyorrhectic debris, crescent-shaped histiocytes, and a notable absence of neutrophils, helping to differentiate KFD from other conditions such as lymphoma and lupus lymphadenitis [14,15].

The exact etiology of KFD remains unknown. Several viral triggers—including Epstein–Barr virus (EBV), cytomegalovirus (CMV), human herpesvirus 6 (HHV-6), and Parvovirus B19—have been implicated, although no consistent causative agent has been identified [6,7]. An autoimmune mechanism is increasingly considered, particularly in patients with positive autoantibodies or those who later develop SLE [16]. This supports the need for long-term follow-up in pediatric KFD patients, especially those with positive ANA titers.

Management of KFD is primarily supportive. Our patient responded well to non-steroidal anti-inflammatory drugs (NSAIDs) and did not require corticosteroids or immunomodulatory therapy [17]. Fever resolved early during hospitalization, and lymphadenopathy gradually regressed. At one-month follow-up, the patient remained asymptomatic, had regained weight, and had no palpable lymph nodes.

This case emphasizes the importance of considering KFD in the differential diagnosis of persistent cervical and supraclavicular lymphadenopathy in children. Early recognition and histological confirmation can prevent unnecessary interventions, such as prolonged antibiotic use, corticosteroid therapy, or invasive investigations for suspected malignancy. Given the potential overlap with autoimmune and malignant conditions, clinicians should maintain a high index of suspicion for KFD, particularly in atypical presentations.

## 4. Conclusions

Kikuchi–Fujimoto disease (KFD) is a rare, self-limiting cause of lymphadenopathy in children that can closely mimic more serious conditions such as lymphoma or systemic autoimmune diseases. This case highlights the importance of including KFD in the differential diagnosis of persistent cervical and, notably, supraclavicular lymphadenopathy in pediatric patients—an especially unusual presentation.

Early consideration of KFD, along with timely histopathological confirmation, is crucial to avoid unnecessary invasive procedures and treatments. Although the prognosis is generally excellent, long-term follow-up is advisable, particularly in patients with autoimmune markers, given the potential association with systemic lupus erythematosus.

This case underscores the need for heightened clinical awareness of KFD among pediatricians and highlights the diagnostic value of lymph node biopsy in unresolved lymphadenopathy.

## Figures and Tables

**Table 1:** Laboratory findings.

Infectious Disease Panel		Blood, Urine, and Stool Cultures: Negative
**Serology**	Viruses	EBV IgM (−), IgG (+, 23 U/mL); Monotest (−); CMV IgM (−), IgG (−); HIV (−); adenovirus IgM/IgG (−); influenza (−).
	Bacteria	Brucella IgM/IgG (−); Rose Bengal (−); Wright test (−); Mycoplasma IgM/IgG (−); Bartonella IgM/IgG (−).
	Other	Leishmania (−); ASTO: 25 IU/mL; Mantoux (−); Quantiferon (−)
	Parasites	Toxoplasma IgM/IgG (−).
**Bone marrow aspiration**		Morphology and cultures were normal.
**Autoimmune/immunologic evaluation:**		Immunoglobulins: IgG 1250 mg/dL, IgM 182 mg/dL, IgA 234 mg/dL, IgE 139 U/mL. Complement: C3 86 mg/dL, C4 25.8 mg/dL. ANA: positive at 1:160 titer. Anticardiolipin antibodies: IgG 6.08 GPL/mL, IgM 7.47 MPL/mL (both negative). ANCA: negative (p-ANCA and c-ANCA). Rheumatoid factor (RF): negative. • Angiotensin-converting enzyme (ACE): 60 U/L (NR: 6–89). • Protein electrophoresis: normal.

## Data Availability

The original contributions presented in this study are included in the article. Further inquiries can be directed to the corresponding author.

## Abbreviations

The following abbreviations are used in this manuscript:

KFD	Kikuchi–Fujimoto disease
ANA	Antinuclear antibody
CT	computed tomography
FNAC	Fine-needle aspiration cytology
SLE	Systemic lupus erythematosus
Kg	Kilogram
USA	United States of America
ESR	Erythrocyte sedimentation rate
EBV	Epstein Barr Virus
CMV	Cytomegalic virus
HIV	Human immunodeficiency virus
ASTO	Antistreptolysine title
ACE	Angiotensin-converting enzyme
ANCA	Anti-neutrophil cytoplasm antibody
IgM	Immunoglobulin M
IgG	Immunoglobulin G
RF	Rheumatoid Factor
IgE	Immunoglobulin E
IgA	Immunoglobulin A
C3	Complement 3
C4	Complement 4
MRI	Magnetic resonance imaging
NSAIDs	Non-steroidal anti-inflammatory drugs
